# Andean Women’s Persistence Amidst Racialized Gendered Impoverishment, Capitalist Incursions, and Post-conflict Hauntings

**DOI:** 10.3389/fpsyg.2022.908673

**Published:** 2022-06-02

**Authors:** Gabriela Távara, M. Brinton Lykes

**Affiliations:** ^1^Department of Psychology, Pontifical Catholic University of Peru, Lima, Peru; ^2^Lynch School of Education and Human Development, Center for Human Rights and International Justice, Boston College, Chestnut Hill, MA, United States

**Keywords:** armed conflict, psychosocial sequelae, Indigenous women, feminist participatory action research, women’s associations

## Abstract

Throughout the last four decades, Andean women from the highland communities of Peru have been significantly affected by ongoing neoliberal capitalist development and patriarchal structures. These intersecting violence(s) took on more horrific dimensions during the Peruvian armed conflict (1980–2000) and have contributed to multiple psychosocial sequelae that linger in the daily lives on these communities as “ghostly matters.” Seeking to face these experiences in a context of ongoing material impoverishment, Andean women from highland communities have initiated multiple associations or economic collective projects. The authors accompanied a group of women who formed a knitting association and facilitated a feminist participatory action research (FPAR), creating opportunities through which these women could engage in action-reflection processes toward enhancing their association. Twelve women from this FPAR process agreed to be interviewed by the first author who sought to explore their understandings about the processes of forming their association in this post-conflict context and the challenges they were facing. Interview transcripts were analyzed using Charmaz’s constructivist grounded theory coding strategy. The findings reveal multiple challenges for women’s collaborative work created by ongoing racialized gendered violence and its intra- and interpersonal effects. Moreover, the findings confirmed that capitalist development dynamics, and, more particularly, resources introduced by agents from outside the community, bring both gains and losses for Andean women. The latter reported having learned skills that allowed them to better insert themselves in a market economy, but that these new activities were displacing more community-based Indigenous practices and traditions. Finally, this study reveals that the wounds created by the armed conflict generated multiple forms of silence that prevent Andean peasants from openly expressing their desires. Despite this, the women in this FPAR process and participants in these interviews are engaging in action-based responses through which they are overcoming some of these challenges and sustaining their association. The article concludes with a discussion of the implications for mental health professionals and activist-scholars working with Indigenous communities affected by armed conflict by underscoring the limitations of interventions based exclusively on the spoken word and arguing for action-based approaches that draw on bottom-up knowledge and practices.

## Introduction

Andean women have faced diverse experiences of racialized gendered marginalization both in their local communities and in the Peruvian society more broadly, within and across time. This marginalization has deep historical roots and can be found in and through contemporary neoliberal, racist, and capitalist systems and structures. The Peruvian armed conflict of the 1980s and 1990s was rooted in these dynamics and affected extensive areas in the southern Peruvian Andes, leading to the death and disappearance of 1,000 of Quechua-speaking *campesinas*^1^ (peasants; Comisión de la Verdad y Reconciliación, 2003) and to complex psychosocial sequelae in their communities (Martín–Baró, I., 1994).

During the past three decades, as one response to this continuum of economic and political violence and marginalization, Andean women have sought to build a better life for themselves and their children. In doing so, they have exercised their agency in complex and sometimes contradictory ways within multiple structural limits and some new opportunities in their socio-historical and economic contexts (Charrad, 2010). Many have opted to come together and organize women’s associations, and in so doing, have found, or been offered, support from both private and public institutions including reparations initiatives, NGOs, state programs, and private corporations. Andean women’s work with these institutions has brought them benefits but also challenges in terms of leaving behind previous campesina cultural practices and values including local ways of being in the world. The research reported here complements a feminist participatory and action research (FPAR) process that was initiated to better understand one these efforts. It focuses on an analysis of in-depth interviews with 12 Andean women from a town affected by the armed conflict and increasing capitalist incursions who participated in the FPAR. These women had decided to work together in a knitting association, seeking a better life for themselves and their families. The first author initiated this FPAR process in 2016, accompanying the knitting association for 18 months as they developed collective efforts to enhance the quality of, and the market for, their knitted products. The second author accompanied at a distance, drawing on years of feminist participatory action research with creative resources in rural Guatemala during conflict and post-genocide processes and in contexts of humanitarian disasters in the US and beyond.

Through participatory workshops that incorporated local practices as well as creative storytelling, dramatizations, and drawings, the authors and the Andean group sought to document experiences of the post-conflict period as well as the strengths and resources through which they could move roughly forward in this changing context. As outsider activist-scholars, the authors facilitated iterative processes through which the women reflected on actions through which they sought to mobilize their individual and collective responses to the sequelae of the armed conflict in a context of ongoing racialized impoverishment. Internal community dynamics and multiple silences and silencing about the armed conflict were reflected in what we saw as the women’s hesitancy to share narratives of these complex experiences. Recognizing that mainstream EuroNorthAmerican psychological and feminist thought tends to equate silence with disempowerment, this article aligns itself with a more nuanced understanding of silence that is grounded in the experiences and activities of these Andean women (Parpart and Parashar, 2019). Moreover, as some Indigenous scholars have noted (see Gone, 2007) and as we have discussed in previous work (see Lykes and Crosby, 2015), situating stressors within historical and sociocultural contexts and/or acting to redress problems is most often a preferred strategy to that of “talking about individual-level or current feelings.” Despite this, the authors hoped that in-depth individual interviews with some of the participants, that complemented time together in the larger group workshops, might facilitate a better understanding of their complex post-conflict gendered and racialized experiences in hopes of also generating meaning making that might enhance women’s broader participation in their community (Lykes et al., 2021). We document below some ways in which these Andean women contested racialized gendered impoverishment in the context of ongoing marginalization and resistance in the midst of what we have come to understand as post-conflict “hauntings” (Gordon, 1997).

This study’s findings confirm the multiple contributions and some of the limitations of participatory, action-based approaches in the wake of armed conflict and structural economic and gendered violence. It begins with a brief overview of the context in which the women’s knitting cooperative was developed, including a discussion of the multiple circulations and intersections of patriarchal, racialized, and capitalist power. We then describe the FPAR processes briefly, situating the in-depth interviews as one aspect of these broader action-reflection processes. After describing the interview protocol, sample and analytic strategies for this study we present our interpretations of the women’s meanings made of their experiences within the knitting association, including their reflections on the limits and possibilities of organizing together in this post-conflict context. We conclude with a brief discussion of limitations as well as implications for future work with rural women in conflict and post-conflict transitions that are continuously constrained by neoliberal capitalist development.

### Andean Women Confront Gendered Racialized Violence

The Andean women participants in this FPAR process are positioned in a rural town within a continuum of patriarchal, racialized, and economic violence that constrains their psychosocial health and wellbeing, factors that persist despite peace accords and transitional justice processes in the wake of the most recent Peruvian armed conflict (Comisión de la Verdad y Reconciliación, 2003). Within these constraints, Andean women continue to participate within local agglomerations of campesina families that have a shared collective history (Urrutia et al., 2019). In these communities, authorities exercise norms that promote traditional cooperative practices that facilitate the administration of the communities’ resources through collective and reciprocal work (Urrutia et al., 2019). However, women—in contrast to their male counterparts—are responsible for tasks both within and outside the home, which renders their work load extremely demanding. Thus, most of the unpaid work in rural areas is assumed by women (Forstner, 2013).

Patriarchal systems and practices configure Andean campesino community life wherein women are subordinated to men. Both at the family and the community levels, men assume leadership positions and women have a subsidiary role, leading to men’s concentration of power and decision making (Diez, 2011; Asensio and Trivelli, 2014). These patriarchal dynamics limit women’s access to land and other livelihoods (Asensio and Trivelli, 2014). Social, cultural, and economic challenges create multiple barriers preventing women from successfully inserting themselves in the socioeconomic dynamics of their territories, rendering many of them dependent on their spouses or other family members. Many women are therefore more vulnerable to multiple forms of violence. In most Andean communities, machismo is entrenched and male domination contributes to gendered family-based violence (Forstner, 2013; Távara, 2019). Moreover, violence against Andean women persists beyond the home as they face constant discrimination in the Peruvian society more broadly due their position at the intersection of racial, class, and gender hierarchies (Babb, 2018).

The most recent Peruvian armed conflict exacerbated many of these dynamics. Although most of those killed and disappeared were men, women from campesina communities lost spouses and sons, or were left behind when men migrated to coastal cities. Many were forced to assume leadership positions in the community in the midst of chaotic times during which they survived multiple forms of sexual violence at the hands of the military and Shining Path. Although the Peruvian Truth Commission (Comisión de la Verdad y Reconciliación, 2003) documented many of these violations including some of the psychosocial effects of forced disappearances as well as forced displacements, scholars and human rights activists argue that violations of Andean women have been seriously underreported and undercounted (Theidon, 2007).

### Psychosocial Responses in Post-conflict Contexts

Although the psychological effects of war and other gross violations of human rights are increasingly noted in transitional justice processes, psychologists’ responding to survivors typically focus disproportionately on individual symptoms, often described as post-traumatic stress disorder (Figley, 1985), decontextualizing them from the familial and community dynamics described above. This individualistic focus often positions women as victims of sexual violence whose only recourse for redress is through legal court trials or individual testimonies before truth and/or reparations commissions (Piper et al., 2012; Crosby and Lykes, 2019). Although this reflects a much-delayed and critical recognition of sexual violence as a crime against humanity (Ellis, 2007), some scholars have suggested that one effect of the growing centering of sexual violence has been the silencing of multiple other structural gendered and racialized violations (e.g., impoverishment and loss of livelihoods and forced displacement), as well as the women’s multiple forms of resistance.

Moreover, transitional justice responses in the wake of armed conflict introduced by human rights activists and psychosocial accompaniers often rely on oral communication, processes unfamiliar to rural campesinas who are disinclined or unaccustomed to discuss openly their thoughts and feelings regarding the conflict and its losses. Furthermore, feminist scholars have pointed out that in cases where survivors are emerging from contested and conflict-ridden contexts, silence is another possible response that should be documented. Parpart and Parashar (2019), among others, have noted multiple meanings of silence, well beyond a lack of speech, arguing that professionals or others who accompany these communities must respect and seek to understand the particularities of diverse acts of silence.

Additionally, psychological responses to war, disaster, and humanitarian crises are typically envisioned as short-term interventions that draw on universalized theories of trauma and recovery (Summerfield, 2001; Lykes and Mersky, 2006). Although those who intervene increasingly recognize the contributions of local knowledge systems and cultural and linguistic practices to healing [Inter-Agency Standing Committee (IASC), 2007], few remain in the field long enough to learn about or engage with such particularities and lived experiences. The second author, among others, has critiqued these short-term interventions grounded in universalized theories and their widespread applications, and engaged in participatory processes through which those who accompany women over time can facilitate the documentation of their experiences rethreading life and community despite a continuum of ongoing injustice and violence (Women of PhotoVoice/ADMI and Lykes, 2000). Other initiatives through which women have resisted these violations include organizing during the early years of the conflict through women’s associations that searched for disappeared family members and demanded justice (Jave, 2014). Others advocated for a means for subsistence for their families in a context of armed conflict, great scarcity, and impoverishment (Reynaga, 2008). The research reported here sought to extend these earlier critiques while documenting women’s resistance through accompanying the Andean women of Huancasancos in the formation of their knitting association, one possible contribution to rethreading life in the context of post-conflict.

### Capitalist and Externally Driven Transitions

The armed conflict emerged and unfolded in a time in which a neoliberal capitalist economic system was rapidly expanding, making incursions into rural campesina communities. During the last decades of the 20th century, the expansion of the market economy increased the number of paid jobs. These changes have partly hindered community relationships based on cooperation and reciprocity, and have promoted individualism among community members (Quispe, 2011). This complex and changing scenario has made rural economies more precarious, and many campesinas, or former campesinas, have found it increasingly challenging to insert themselves into these market dynamics. The situation for women is made more difficult due to the structural limitations described above, and thus, the limited resources available in rural economies are unequally distributed between men and women (Asensio and Trivelli, 2014; Babb, 2018).

Another significant change that occurred in the last years of the conflict and in the post-conflict period in rural campesino communities was the greater presence of external agents both from NGOs and from the state. Moreover, the Peruvian transitional justice processes post-conflict led to the implementation of various reparations programs. Many of these collective reparations programs were implemented through economic development or productive projects that brought together groups of victims, including women, in activities through which they sought to create a livelihood, such as animal rearing or craft production. Researchers have documented some of the challenges of collective reparations projects, particularly vis-à-vis the lack of continuous technical and economic support by state agents, and the mistrust community members have felt among themselves, a psychosocial sequel of the conflict that often undermined their capacity to work together in projects where significant material resources were involved (Bunselmeyer, 2020).

NGOs have also had an important presence in rural areas in the years following the conflict through the implementation of economic development projects, many of which have targeted Andean women particularly through craft production (Ruiz-Bravo, 2005; Forstner, 2013). These projects have provided monetary income and technical training, including in topics related to women’s rights and gender equity, and provided a female exclusive environment where women could develop bonds among each other and strengthen personal and relational skills while developing self-confidence (Forstner, 2013). Similar to post-conflict situations in other countries (see Yadav, 2021), the Peruvian post-conflict situation in rural communities created scenarios wherein Andean women were able to benefit from resources provided by external agents such as those described above. Andean women of the 21st century are more likely to have greater practical skills, better health, more education, and more awareness about their rights than Andean women from any previous generation, conditions that have increased their chances of providing a better life for their families (Asensio and Trivelli, 2014).

Despite these improvements, Andean women in post-conflict contexts still face multiple challenges. Some of these are related to patriarchal gender systems that are more resistant to change and prevent women’s equality. Others reflect the ways in which external professionals working with Andean women often facilitate neoliberal capitalist models for income generation that clash with people’s previous indigenous values, beliefs, and practices. Such conflicts sometimes lead to the devaluing and desertion of previous ways of being in rural communities. Below we document how the group of Andean women with whom the authors collaborated engaged and responded to these multiple systems of structural oppression and limited opportunities as well as some of their reflections on the actions they have taken in the formation as well as maintenance of their knitting association, one act of persistence despite the odds.

## Materials and Methods

### Participants in Context

Twelve Andean women from the town of Huancasancos in the region of Ayacucho in Peru participated in this study. The population of Huancasancos at the time of this study was approximately 3,000. Most families are campesinas and are dedicated to farming activities and to their small businesses (Instituto Nacional de Estadística e Informática, 2018). During the past decade, extractivist industries began work on the outskirts of the town, which is located 12,000 feet above sea level, while also approaching diverse community groups, seeking to contribute economic resources that might mitigate objections to their incursions.

Prior to the work described herein, members of the Shining Path (SP) had entered the town and gained support among the younger generations (Comisión de la Verdad y Reconciliación, 2003). When the SP took control in the early 1980s, they killed many of the town’s authorities and sought to control the community’s material resources, acting in violent ways, and attacking anyone who opposed their methods. After several months, the military arrived, engaging in bloody confrontations with the SP. Although the latter’s members deserted the town after these initial encounters with the military, sporadic violent confrontations persisted for another decade (Comisión de la Verdad y Reconciliación, 2003). As noted above, the Peruvian truth commission (CVR) was established in the wake of the armed conflict and charged with investigating the crimes committed during this period. The CVR identified 100s of people in Huancasancos who had lost their lives, been disappeared, or been injured, and many others who had lost the few material possessions they had before the conflict (Comisión de la Verdad y Reconciliación, 2003). A reparation program was implemented granting, among other measures, individual reparations in the form of payments to surviving family members, and collective reparations in the form of economic-productive projects.

Many townspeople have strongly criticized both of these efforts at redress. They found the criteria for qualifying as a victim entitled only some survivors to individual reparation payments and that collective reparation projects failed due to the lack of continuing support by the state (Ulfe and Malaga, 2021). The knitting workshop space that participants in this feminist PAR project were using was initially part of a collective reparation project. Local informants noted that the project had been abandoned when the funds provided by the initial program ran out and the group was not willing to invest its own resources. With the support of a community-based worker from a local mining company, the women from the knitting association were granted permission by the municipality to use the space. The mining company also provided technical support and training to the emerging women’s association and facilitated the first author’s introduction to them.

As described above, 12 women from the FPAR process that the first author had been facilitating were interviewed. Their approximate ages were between 30 and 65. All had children and most were married or had a partner; only three were separated and one was a widow whose husband had been killed during the armed conflict. All participants come from campesina families and thus, most were dedicated to a combination of farming activities, selling their home-knitted products, and working in small family businesses, such as shops that sell basic goods in the town when they joined the association.

Most of the participants were children, adolescents, or young adults during the worst years of the armed conflict. They reported vivid memories of these violent events, remembering the killings and confrontations that took place in the town. Some had family members who were killed or disappeared. Although they timidly shared these experiences during the workshops, participants did not discuss their engagement with either side of the conflict either during the workshops or in the in-depth interviews. We argue here that memories of the conflict linger as “ghostly matters” (Gordon, 1997) alongside harsh economic and political realities whose constraints often contribute to campesinas living silently side-by-side with former foes in order to survive. As discussed above, these realities create challenges for human rights activists, psychologists, or humanitarian aid workers who rely heavily on the spoken word as a vehicle for providing resources that are designed to enhance wellbeing and contribute to healing fragmented community ties as they seek to accompany these communities in processes through which they seek to create better lives.

### Documenting the Spoken Word as Resource and Its Limitations

The core of this FPAR project included participatory workshops whereby the first author and the participants engaged in action-reflection processes toward the collective construction of knowledge using creative art-based techniques and embodied practices (Távara, 2018). We sought to create a space in which the women could reflect on the actions they were taking as they sought to develop a viable, collectively organized knitting association. As discussed above, the individual interviews with the 12 participants analyzed in this study were initiated during those18 months of fieldwork in which the first author visited the community regularly, seeking to develop a relationship of “just enough trust” (Maguire, 2001) with the participants and other community members to accompany them as they resisted some of the multiple effects of the circulations of power that marginalized the campesina women of Huancasancos.

The guide for these interviews, conducted between July and December of 2017, was developed collaboratively by the authors to further explore the women’s experiences of collective work and their perceptions of its benefits and challenges. We also sought to better understand the current situation of women’s associations in the town, and how machismo and everyday forms of racialized gendered violence impacted Andean women’s capacity to overcome legacies of distrust and work together as they searched to improve their and their families’ lives. In this way, the interviews constituted an additional space where the women could reflect individually on their participation in the workshops and the broader collective processes.

Given that memories of the armed conflict, its psychosocial sequelae in the community (e.g., feelings of mistrust and fear, among others) and also current worries and misunderstandings among association members had proven difficult to address with the women in the collective workshops and seemed to be limiting the association’s progress, the interviews were designed to offer accompaniment that might facilitate increased meaning making of diverse experiences and understandings of both past and present challenges. As suggested above, rural campesinas are less likely to engage in oral processes that require them to share personal experiences; thus, interviews may be limited in facilitating healing of wounds from fragmented community ties, such as those the women were facing. Despite that, when accompanied by actions through which the women could mend these ties, the spoken word offers a resource, familiar to EuroNorthAmerican-educated researchers, for collaboratively reflecting with the campesinas about their actions as they seek to build a better future.

All the interviews were conducted by the first author in Spanish. Although this is the second language of all participants whose first language is Quechua, they felt fluent enough to express themselves in Spanish. A bilingual Quechua interpreter accompanied the first author during the interviews in case interpretation was required. This young female professional from Ayacucho also contributed to the facilitation of the workshops; thus, she was familiar with the group of women. Only two of 12 participants expressed themselves briefly in Quechua, sharing an idea or two for which they could not find the words in Spanish. The rest of the interviews and the interviews of all other participants were conducted entirely in Spanish.

The interviews took place either in the women’s homes or in a private space chosen by them. Before each interview, the first author presented the informed consent, explained the goals of the interviews within the broader FPAR process, and addressed any questions that the participants had. The presence of the Quechua interpreter throughout this process allowed the researchers to address any misunderstanding created by language barriers. Interviews were then transcribed by both the first author and the Quechua interpreter and were analyzed by both authors. The Boston College’s Institutional Review Board approved the broader FPAR project of which this study is part.

As mentioned above, the individual interviews were designed to offer an additional space for dialogic relationality that might better facilitate the co-construction of knowledge with the women, campesinas who typically spoke little of their personal experiences. The subject position of the interviewer vis-à-vis the participants affects the processes that take place therein and informs the data analysis and interpretation processes. Our subject positions as researchers facilitate and constrain these multiple processes of co-constructed knowing. As a Peruvian upper-middle class mestiza, the first author’s privileged background has allowed her to benefit from the circulations of power present in the Peruvian society and to be educated in the global north. The second author is a highly educated, white upper middle-class academic from the US who continues to benefit from white supremacy and hegemonic EuroNorthAmerican circulations of academic power. She has worked for nearly three decades with Indigenous women and children in the majority world and with transnational migrant families, seeking to accompany them in their struggles through pragmatic solidarity. Both authors are academic-activists who continuously critically interrogate how our subject positions inform and constrain our understandings of Indigenous women’s meaning making processes.

### Data Analysis

The analysis of the interviews was informed by constructivist grounded theory (Charmaz, 2014). This type of analysis is underpinned by line-by-line coding which allows the researcher to stay close to the data and to look across participants from the initial stages of the analysis. It facilitates a process by which the researchers can capture the meanings made from the bottom-up, creating increasingly more abstract levels of analysis, while staying grounded in the data (Charmaz, 2014). Using Nvivo, a qualitative data analysis software, the first author analyzed the interview transcripts and created approximately 1,700 first level inductive codes. These first level codes were then revised and grouped into 18 axial codes which reflected the shared meanings they contained. The creation of the axial codes took place through an iterative process in which the first author, in consultation with the second author, adjusted the axial codes based on how well they were able to capture the first level codes grouped under them and how well they reflected the meanings made by all participants. Thus, while creating these codes the authors had to continuously go back to the first level codes and the interview transcripts, seeking the best fit. After the 18 axial codes were created, they were organized in two sections that comprehensively represent the women’s meaning making processes and respond to the research focus.

## Andean Women’s Understanding of Their Current Situation: Seeking Changes in the Midst of Machista and Capitalist Challenges

The following presents two sections in which we analyze the in-depth interviews generated with these participants. This first section explains Andean women’s understandings of their current situation and includes the changes participants saw in women’s work as well as their understandings of how gendered racialized violence and capitalist dynamics were affecting them.

### Changes in Andean Women’s Work

Participants narrated changes taking place regarding women’s work in their town. They explained that women had been previously only dedicated to farming and domestic activities. However, in the last decades, they had progressively started working outside of the home as well, mostly in small family businesses—a movement that had come with challenges. They explained that many women had great difficulties balancing their responsibilities inside and outside the home and had experienced considerable stress. Furthermore, many women described feeling guilty for working outside their homes believing it to be associated with neglect of their children. These transitions can be better understood by analyzing participants’ ideas about family and women’s roles and place within the family.

Most of the participants upheld the importance of the traditional nuclear family and said it was important to protect it. Moreover, they explained that within the family the women are responsible for the children and for all of the domestic chores, as well as to attend to their spouses. Women are the “cornerstone of the home” because it is “in their nature” to be caring and hardworking, participants said. As noted above, these beliefs are held in Andean communities more broadly, both by men and women. Therefore, even when Andean women are progressively seeking to expand their daily activities by working outside the home, underlying beliefs about their roles within the family might be undermining their efforts.

### Machismo and Gendered Racialized Violence

For participants, machismo is very present in Huancasancos. They explained that machismo is expressed through intimate partner violence, both physical and psychological. Some participants mentioned that physical violence was slowly decreasing while forms of psychological violence persisted. They described how men mistreated and humiliated their wives, doubting their capacities and mocking them. Some men also controlled their wives by not letting them leave the house, go to meetings, or work outside the home and, if they left home, they were questioned about their whereabouts upon their return. As one woman mentioned as: “they make them report back, where are they going, what for, until what time, with whom,” Some men also control women economically, because they do not have paid jobs and men control all the monetary income of the house. In this regard, participants described how some women have stayed “like children,” submissive to their husbands needing them to tell them what to do.

Other women noted that machismo is expressed through structural violence. These included obstacles preventing women’s higher education, particularly when they sought to pursue a male dominated field. Other structural obstacles prevented women from assuming leadership positions and/or becoming community authorities. One participant mentioned as: “There are no women who go out, who develop, we do not see that, to say ‘hey look, how she is doing it’. There are no examples to follow.”

Despite this discouraging scenario, participants explained that Andean women have begun to resist machismo, contributing to the perceptions of some of those interviewed that it is decreasing. They explained that women increasingly acknowledge forms of machismo in their daily lives. Others described denouncing violence against them by going to the police station or to other state offices put in place to protect women. Other women noted that they had faced and stood up to their husbands and demanded to be treated with respect.

Racism and ethnic discrimination intersect with machismo. Many participants explained how people tended to mistreat and discriminate against Andean women who used traditional skirts and were monolingual Quechua speakers, being called “*polleronas*,” an allusion to the skirt or “*pollera*.” These women are perceived to be poor and less educated. Participants described that oftentimes people mocked them because they did not speak well, “Maybe they make mistakes when they talk, they fail. Then people start whispering behind their backs. That is the humiliation.” Participants also explained that sometimes professionals who work in state offices in the town discriminate against Andean women, treating them poorly. However, other participants said that there was no discrimination or racism in Huancasancos because all were countryside people, campesinas, and thus, they were treated equally.

Participants explained that both racism and machismo had profound effects on women. They narrated how many Andean women are inhibited, fearful, and insecure because of how their partners treat them. These feelings made them anxious about speaking in public or about expressing their opinions with others whom they did not know well, rendering them very self-conscious. Furthermore, participants explained that this inhibition and insecurity made other people, or their spouses, mock Andean women or mistreat them even more, creating a vicious circle. Participants also mentioned that in some cases, women held back from trying new things or embarking on new projects because they feared they would not be able to learn. This sometimes led them to give up on their aspirations and put others’ needs before theirs. Along this line, participants described some women as being “adrift” and settling with what they have.

Machismo also prevented women from collectively organizing. Participants explained that some men did not want their spouses to gather and work together with other women or go to meetings, saying that they only went to gossip. Furthermore, men were described as getting angry at their spouses when they gathered in women’s collectives because they said they were neglecting their responsibilities at home. Participants described how this constituted an obstacle for women who sought to work together, for instance, in their knitting association, because some preferred not to attend these meetings in order not to upset their husbands. Despite this obstacle, participants emphasized that working together as women and gathering was important as it could help women to counter machismo.

### Capitalism’s Growing Influence

Another important element to understanding Andean women’s current situations are the changes brought by the increasing influence of capitalist development. Participants described how their town had changed rapidly in the past years. They explained that there used to be very few businesses that sold basic goods brought from outside the town and that these had been in great demand. Currently, there are several businesses and also more paid jobs which allow people to buy goods in these businesses. Participants went on to explain that because of these changes people in the town expected others to pay them for things that in the past they did as volunteers or through the reciprocal exchange of work. Women now hired other women to help them care for their children, and families hired people to cook at their parties. They explained that in the past families who were close or part of the extended family would support each other voluntarily in these tasks, while now these reciprocal practices are being lost.

As can be seen, Andean women in Huancasancos have gone through multiple changes during the past several decades. They noted that in addition to the situation of vulnerability and inequality generated by machismo and racism, capitalist dynamics pressured women to secure forms of monetary income. In this scenario, and facilitated by the presence of ideas brought from outside the town by professionals—for instance, from the mining company—a group of Andean women sought to create a women’s knitting association that could provide an opportunity to better the future of themselves and their families.

## One Andean Women’s Knitting Association: Responding to Current Challenges

This second section of findings presents the Andean women’s response to their changing context and the challenges they were finding along the way, particularly those related to translating their previous skills and knowledge(s) and the interpersonal difficulties they were encountering. The section explores participants’ decisions to persist in their knitting association despite these challenges.

### Translating Previous Skills and Knowledge(s)

Most of the participants openly expressed enthusiasm and motivation to work in an association. In talking about that process, they narrated challenges they were finding along the way and more of which they feared encountering in the future. One such challenge related to their lack of the knowledge and skills needed to run or be part of an association, affirming that this way of organizing was a new experience for them. As one participant expressed, “I would like someone to explain to me about organizations, how, why…Because we are in this organization, but at least I do not know about organizations.” Participants’ comments during the interviews conveyed that Andean women have an important set of skills and knowledge(s) related to their agricultural background; however, they did not perceive that these skills and knowledge(s) would easily translate into the skills required to face these new tasks.

Participants shared how most members of the association are women that come from families that have been dedicated to agriculture and cattle rearing, and they have produced goods mostly for their own subsistence or for trading or selling on a very small scale, that is, not for commercial purposes. Furthermore, participants explained that to conduct these farming tasks, families in the community have relied on principles of reciprocity, including taking care of each other’s animals. Also, they explained that when working the land, Andean families have certain practices that seek to stay in balance with nature and respect its cycles. They feared that these principles of balance and reciprocity were quite different from the principles underlying businesses in a capitalist economy where the goal is to produce more and compete with others for individual advantage.

Despite these contradictions, knitting represents a set of skills and knowledge that Andean women have maintained over time and have brought into the present. Participants explained that for many generations women have worked animal fibers and have used them to produce clothing through knitting. In previous generations, this was done using natural fibers and spinning them into yarn with a *puchka*, a traditional spinning needle. As one participant mentioned, “Before we used to make our own threads, we used the puchka to make sheep wool yarn, llama wool yarn… everything.” Although in the present there are not many women who still practice this traditional technique (in the association only one of the older women still used a puchka), many women in the town still knit. The group of women who initiated the association had sought to harness that knowledge and organize this women’s knitting association, with an expanded agenda of generating income.

The knitting association is, to a great extent, framed by the participants as a business. Most described this association as a business that could help members from the town. As one participant said “… I want [this knitting association] to progress, the younger women who are in it, they have its future, tomorrow they can have a business. They have to have the idea of forming a business, at least a little one, right? That way they can improve their daily life.”

This understanding of the knitting association can partly be explained by the influence of outside professionals from the state, private companies, and NGOs, working with town members. As mentioned previously, most of these professionals work in institutions that are focused on training people from Andean communities in skills that can allow them to produce and sell so that they can better insert themselves in a neoliberal capitalist society. Despite acknowledging the losses generated by the influence of capitalism, participants recognized their need to insert themselves in the market economy and thus, many of them expressed great appreciation for these trainings. Therefore, the formation of the women’s knitting association responds to, on the one hand, the women’s interests and traditional skills in knitting, and on the other, to the influence of a capitalist system and the women’s needs to make ends meet, even when this might mean sacrificing previous ways of living.

Some of the women in the knitting organization had previous business experiences, including having worked in small family or personal businesses, e.g., in stores that sell basic goods or booths at the local market that sell yarn and knitting utensils or food. Although they had gained skills from these past business experiences, they perceived that they lacked skills to apply them in the knitting association. The knitting association, in contrast to their earlier, family-based experiences, was developed across families and as a collective endeavor where the assets belong to the association and not to individuals. Participants explained that in the association all women had to contribute with their work and with investing initial seed money. Some mentioned that providing this initial monetary investment was challenging, either because they did not have enough money or because they did not want to take the risks of investing it. They also acknowledged that when money is involved, mistrust can emerge more easily. Moving forward with a collective endeavor required trust in other members and a greater commitment to the project. In light of some of the obstacles of an interpersonal nature, they had previously noted as described above, many found this challenging.

### The Interpersonal Challenges of Working Together in Post-conflict Times

The participants of this study were very self-critical about the interpersonal challenges women in their town faced and about the need to heal complex wounds in their relationships with each other. They recognized how several aspects of their collective and personal histories, such as the armed conflict and their continuous experiences of gendered racialized violence, had an impact on their capacity to connect with each other.

Participants mentioned that one of the main relational challenges they faced was their capacity to trust one another. They explained that this mistrust was expressed through their fear of what others would think about them; some noted that they were very self-conscious and also feared being treated poorly. They saw this mistrust as related to previous and ongoing experiences of violence and abuse that Andean women experienced, either in their families or beyond, noting that these experiences create anxieties about future relationships. As one participant mentioned, “Women who are mistreated are fearful, for example, when they ask something. Since their husband mistreats them, they think that maybe others will mistreat them as well (…) They think that all are the same, right? That they can yell at them, or that they will not teach them.”

This mistrust was associated with previous negative experiences in other associations in the town. Participants explained how these previous associations had failed because some members took advantage of others, cheating or appropriating the resources and goods of the association for themselves. One of these projects was a collective reparation granted to one of the neighborhoods to set up a chicken farm. Participants explained how 1 day the chickens disappeared, leaving the members of this group in shock and with a strong sense of mistrust. Participants mentioned that experiences like this affected women’s capacity to trust others, believing that this demonstrated how people could be dishonest and selfish.

Women also perceived that their lack of familiarity with other women in the town, despite having lived in Huancasancos practically all their lives, hindered their trust of one another. Participants explained that due to this “lack of closeness” they did not feel very comfortable expressing themselves in meetings of the association and believed this self-silencing could be leading to difficulties in communication and even misunderstandings. For example, one participant mentioned, “If there is a woman who does not talk because she is humble and shy, maybe she is like that, but others will say ‘she does not talk or say anything, she must be angry’.” Participants believed that this lack of openness in some members could create difficulties in openly discussing issues about their association and how to move forward with their projects.

Feelings of resentfulness among women were also noted as fragmenting relationships among members in the association. They explained that sometimes women could be resentful and “feel bad” when they saw other women progress. These feelings sometimes kept them from providing support and helping each other. Many of the participants perceived that support between women has decreased in the town in the past years and they worried that this would constitute an obstacle for their association.

The armed conflict profoundly affected many of the women in the association. One participant even noted that this violent period had left many in the town “psychologically wounded” and that they continuously felt the effects of the conflict. Some participants feared violence would return. They explained that because of this fear they had learned to stay silent and not talk openly about the conflict, nor speak up when they saw something in the town that seemed wrong or suspicious. As one participant said “We kept quiet. I have stayed like that until now. I do not speak anymore; if I see something I do not speak about it. (…) I have also taught my children [that] ‘if you go somewhere, you are not going to talk, you better stay quiet’.” Self-silencing during the armed conflict may have reinforced current local tendencies not to speak openly about their feelings. Many feared that speaking would have associated them with one side of the conflict or the other, whereas silence might be perceived as neutrality.

Participants explained that people in the town also felt resentful, and even angry, when they remembered the armed conflict, particularly toward those who people perceived as having supported the Shining Path. The reparation program implementation also deepened feelings of anger and resentment in this town. Some women perceived that reparations were being granted and distributed in an unfair way, explaining that this was further damaging the relationships among people in the town. Thus, as can be seen, feelings of anger and resentment seem to be lingering among members of this community, and some participants perceived this as having an impact on the relationships among those in the association or others who might have wanted to join it.

Despite the above, these participants found it extremely difficult to talk openly about the armed conflict and they noted further that most in the town preferred not to do so. The women in the FPAR workshops had explained how painful it was to remember those years and how they felt that remembering them would hold them back and not allow them to move forward. However, they also mentioned how the violence of the conflict was impossible to forget and how their memories and fears repeatedly come back to them. One woman mentioned, “When I went up to the mountains on my own, when there were no cars, I said ‘what if from that cave comes out a terrorist and kills me. Maybe he is living in that cave and I cannot see him, maybe he is looking at me now.’ So, it’s a fear we have.” The armed conflict constitutes an ever-present past whose hauntings continue to circulate among participants in the midst of self and other-imposed silencing that are perceived as eroding community ties and disrupting possibilities for future collective work. Relational difficulties that have emerged from it are threaded through the community’s daily life. Yet, within that context, opting for silence may also constitute a proactive strategy for facilitating possibilities of working together. Below, we explore some of the ways in which Andean women persisted in forming a knitting association in the midst of these complexities.

### Keeping the Knitting Association Afloat: Andean Women’s Persistence

Despite the many challenges, Andean women participants perceived as they worked to initiate and develop their knitting association, they persisted in this endeavor. We noted—and many described—examples of their strategies that contributed to building and sustaining the association. Some narrated stories of sacrifice, of having endured great suffering and losses of family and friends. Most shared how they had started working when very young, either in the field or selling things on the streets of the town. Some narrated how, in their search to make ends meet, they had worked in jobs considered for men (given the demanding physical requirements) and had performed them successfully. Because of these experiences many of the participants believed that they, and women in general, were resourceful and had the ability to solve problems and find alternatives despite their fears if they were determined to do so. As one participant said “even if you are nervous or afraid, you say ‘it does not matter, I have to do it’.” Participants had also witnessed how their past efforts have yielded fruits; they described how by working hard they had been able to build their homes or set up their small family businesses. Many of these efforts had been undertaken to support their children and their families. Participant’s narratives suggest that these experiences of personal sacrifice and effort informed the multiple ways in which they approached the challenges of forming the women’s association.

Their interests, desires, and aspirations for the future appear to be another element supporting participants in their collective project of running the knitting association. Despite having lived through adversity, participants were able to articulate goals in the midst of taking actions for themselves, their families, and for women in their town. They explained that women in their town liked to, and knew how to, knit and that they knitted well. Moreover, they emphasized that women could continue learning about knitting and other skills and topics and that they could work to move forward. Furthermore, participants identified many qualities in the women in the town, explaining that some were determined, responsible, mature, and resourceful. They noted leadership skills in some, clarifying that a few had even occupied important positions in the town. They explained that the association needed that type of leadership.

### Recognizing Benefits From Women Associating With Each Other

Despite the many challenges noted regularly, participants identified benefits and strengths of a women’s association. They explained that by working in groups, women learned and taught new things to each other, not only about knitting but about life in general, providing guidance, and giving each other advice. As an association they could invite professionals outside their group to train them in new skills, for example, about women’s rights. As one participant mentioned, “By being in an association, we can know more about our rights (…) talking with others, we can clear our doubts [about] what is good, what is bad. I used to be all the time up in the mountains and caring for my children, so I did not know.”

Participants also explained that when women worked in groups they dared to do more things because they encouraged each other to do so. They hoped that through these processes they would enhance their self-esteem and recognize what they could accomplish. Gathering in the association also helped women feel less stressed or unhappy because they could be with other women and step outside of their homes, disconnecting from their domestic chores, at least for a while. As one participant said, “Working in groups, among women, we feel better because we can leave the stress of our homes for at least a while, and be with friends.” It seems that being part of a women’s group can bring important benefits to women and support their wellbeing as well as their personal and collective development.

Participants also emphasized how, through their association, women could combine their strengths and abilities in knitting, knit in greater quantities, and respond to bigger orders for their products. They hoped that this would allow them to generate monetary advantages for themselves and thus create changes in their families’ dynamics; they sought to provide for their children and not be dependent economically on their husbands. As one woman mentioned, “There is a lot of work here, they come asking [for knitted products]. So, we call other women to knit as well. That way we do the job more quickly and (…) at least women can take home a little bit of money for their children.” Moreover, for some participants, this first women-only association in the town was proof of what they as women were capable of doing. These participants felt encouraged to see other women interested in joining the association and wanting to work in and be part of it. For them, this revealed that women liked to work together, encouraging their belief in the potential of the association.

Others persisted in the women’s knitting association as they appreciated it as providing new ways in which women were participating, both at an individual and collective level. Since they had started the association, women had begun to “open up a bit”; they now can speak more easily in the group and voice their opinions because they have the opportunity to practice in the group. Being less submissive was evidenced through their going out of their homes and meeting with other women. Some noted that women have “conscientized” and have “opened their eyes,” women now want to change. They explained that these changes are fairly recent, and some say they might be brought by the younger generations, because in the past, women did not talk about working and participating in groups of women. In the past, women were quiet, stayed at home and did not know their rights. Now, women wanted to work with other women, organize, and have more freedom.

## Discussion and Concluding Thoughts

This study reveals how significant changes have taken place in the lives of Andean women in this rural community as they progressively positioned themselves beyond the home, in the public sphere as economically active agents. However, in this process, women were encountering persistent and new challenges. Among the former was racialized gendered violence in the form of machismo reflected in the ways women were mistreated and abused by their husbands, and in the profound intrapersonal wounds created in them. Importantly, as many noted above, these asymmetrical gender relationships were also sustained by Andean women themselves as reflected in the ways in which they positioned themselves in traditional gendered roles within the family. For many Andean women from rural communities, a significant part of their identity is founded on the belief that they have a central role as mothers and care-givers, a role that many women have come to strongly value and from which they derive great strength (Ruiz-Bravo, 2005; Reynaga, 2008).

This study confirms that these beliefs are becoming more complex as some women realize that they are also capable of working outside of their homes, a change that will contribute to their independence from their spouses. However, women noted tensions they experienced as they sought to work outside their homes while sustaining ideas about their role as the “cornerstone” of the family. Despite the benefits, their campesina identity has provided for Andean women; in this changing context, it has also brought several challenges, with some noting that they found it difficult to share or let go of these tasks and roles in the home while seeking to be part of the association. Thus, change is both a resource and a challenge, and for some among the older generation, letting go of being a “cornerstone” within the family is often perceived as too steep a cost for participating in a neoliberal capitalist association.

EuroNorthAmerican-educated feminists have often times criticized this essentialized identity of women as mothers and care-givers, perceiving it to limit women’s protagonism or their capacities to struggle for women’s equality and liberation (Boesten, 2010). Recognizing the importance of contesting essentialized identities that might be supporting Andean women’s oppression, it is also important to acknowledge that changes in Andean women’s identity and roles must be driven and configured from the bottom-up, that is, by Andean women themselves as active agents. As recognized by the participants, these changes in socially gendered dynamics have already begun, facilitating women’s agency on the one hand, while also giving place to losses on the other (Charrad, 2010). The exercise of their agency, in and through these changes, require Andean women to decide for themselves what aspects of their previous identities they leave behind, which ones are transformed and what new aspects are incorporated into how they position themselves within and beyond the family. As outsider feminist activist-researchers our role is to accompany Andean women as they enact these changes while facilitating spaces in which they can reflect on them and, in this way, decide how they seek to move forward.

As seen in this study, changes in Andean women’s lives are also being brought by capitalist dynamics many times promoted by outside professionals and their interventions. The relationship and work conducted with these professionals have brought several benefits to these Andean women, including, among others, knowledge about women’s rights and training in skills through which they enhance their self-confidence. However, this work has also brought—knowingly or unknowingly—new forms of interaction among people, influenced by capitalist dynamics, that are perceived by the participants as having detrimental consequences for Andean women both at individual and collective levels. These new ways of interacting require the ability to produce and sell goods or services that are valued in a capitalist economy. They have required the participants to acquire new abilities and skills, many of which they value. However, they have also required them to leave behind previous ways of being in the world and with others rooted in their Indigenous and campesina identities. Participants are aware of these losses and sometimes feel that their Indigenous knowledge(s) and practices are being devalued. However, they are also aware of the need to adapt to these new market logics in order for them and their children to have an opportunity to better respond to their material needs.

Change is inevitable and will continue to take place in Andean communities; their members will continue to acquire new skills, knowledge(s), and practices—many of them informed by capitalist dynamics—as they leave others behind. However, these processes of systemic change should by informed from the bottom-up by Andean communities. To this end, as outside mental health professionals and activist-scholars it is important to facilitate processes in which Andean women and their communities can reflect on the actions they are taking vis-à-vis these changes, and how they want to respond to them. In this way, Andean women, particularly those of the younger generations, and their communities might have greater chances to inform these changes and engage with outside professionals in ways that advocate for change in the way they wish to see it.

This study presents some limitations, including a small sample which means that the findings may not generalize to processes in other post-conflict Indigenous contexts. Moreover, language differences also place significant limitations given that the first of language of participants and researchers differ and the findings were analyzed in Spanish, the first language of the first author, but were written in English, the first language of the second author. These linguistic complexities are challenging and some of nuances of the meanings co-constructed by participants and researchers might be lost in translation.

The limitations presented above add to those discussed throughout the article about the spoken word as a means for redressing the wounds created by armed conflicts in Indigenous communities. The findings presented herein place at the forefront a significant challenge for mental health professionals and activist-scholars who work with these communities. These professionals need to find alternative ways of accompanying individuals’ and groups’ healing processes that do not rely exclusively on the spoken word while also being challenged to reconceptualize dominant understandings of silence as a constraint and of talking as healing. For women in this study, like many Indigenous women in other post-conflict situations (see Crosby and Lykes, 2019), silence in lieu of talking among each other or about the armed conflict and its psychosocial sequelae is frequently a dominant or preferable strategy. As explained above, memories and feelings created by the violence of the conflict linger as ghostly matters with limited possibilities of being openly discussed. However, this should not be interpreted as a failure to heal or to move past previous fragmented ties. As argued by Parpart and Parashar (2019), in contested social terrains, silence can be a creative way to adapt to difficult circumstances and can become a source for healing. Along these lines, we argue that the participants were opting for actions over words as they sought to mend these fragmented ties and work through previous conflicts. We suggest here that the women perceived the formation and persistence of women in their association as one such action, as a way to invest in and bet on their capacity to rethread life and move forward as they seek to heal together from past and ongoing racialized gendered violence and impoverishment. Thus, their silence should not be interpreted in isolation but rather as accompanied by their performances through which they sought to create change and enhance wellbeing in their post-conflict community (Parpart and Parashar, 2019). As mental health professionals and activist-scholars who are interested in working with these communities, our challenge is to recognize these alternative ways of healing through action when they occur, while creatively envisioning ways to accompany and strengthen them.

## Data Availability Statement

The datasets presented in this article are not readily available because per requirement of the Boston College IRB, the data collected for this project must be destroyed by November 2022. Requests to access the datasets should be directed to gtavara@pucp.edu.pe.

## Ethics Statement

The studies involving human participants were reviewed and approved by the Institutional Review Board, Boston College. The participants provided their written informed consent to participate in this study.

## Author Contributions

GT collected the data, conducted the fieldwork for this study, including the conduction of individual interviews, consulted with MBL throughout the data collection and fieldwork process to make decisions regarding it, and with regards to the data analysis, she conducted the first level analysis of interview transcripts in consultation with MBL. GT and MBL conducted the second level analysis and the creation of the axial codes, wrote the manuscript, and designed the individual interview guides. All authors contributed to the article and approved the submitted version.

## Funding

Part of the funds for this study was granted by the Pontifical Catholic University of Peru, through their “Fondo de Apoyo a la Investigación - FAI,” grant FAI-0036-2021.

## Conflict of Interest

The authors declare that the research was conducted in the absence of any commercial or financial relationships that could be construed as a potential conflict of interest.

## Publisher’s Note

All claims expressed in this article are solely those of the authors and do not necessarily represent those of their affiliated organizations, or those of the publisher, the editors and the reviewers. Any product that may be evaluated in this article, or claim that may be made by its manufacturer, is not guaranteed or endorsed by the publisher.

## Footnotes

^1^We use the word *campesina (s)*, instead of *campesino(s)*, as a translation of peasant throughout the article. We have chosen to use the feminine version of this word in Spanish given that this article is about peasant women.
